# External cervical resorption treatments in routine dental practice: a nationwide registry-based cohort study with long-term follow-up

**DOI:** 10.1186/s12903-026-09359-3

**Published:** 2026-07-23

**Authors:** Fernando J. Mota de Almeida, Jonas Norberg Cedering, ENDORECO Collaboration Group, L. Bjørndal, VS Dawson, H. Fransson, F. Frisk, P. Jonasson, T. Kvist, M. Markvart, M. Pigg

**Affiliations:** 1 Department of Endodontology, Institute of Odontology, The Sahlgrenska Academy, University of Gothenburg, Box 450, 405 30 Gothenburg, Sweden; 2 Public Dental Service Norrbotten, Tandvårdens Kompetenscentrum, Norrbotten County Council, Box 922, 971 28 Luleå, Sweden

**Keywords:** External cervical resorption, Root resorptions, Registries, Outcomes assessment, Prognosis, Survival analysis

## Abstract

**Background:**

External cervical resorption (ECR) is a dental condition characterized by irreversible loss of tooth structure due to periodontal tissue activity, with varied treatment approaches. Evidence on outcomes mainly comes from retrospective studies in specialized settings with limited follow-up, whereas population-level real-world data are scarce. The Swedish National Dental Health Registry (SNDHR) provides a unique opportunity to examine treatment patterns and long-term outcomes of ECR at a national level. This study aimed to map treatment modalities used for teeth diagnosed with ECR in routine dental care and to assess tooth survival over extended periods, as well as to explore associations with available patient- and treatment-related factors within the constraints of registry data.

**Methods:**

Data from the SNDHR between 2009 and 2018 were used to identify adult patients with ECR and associated treatments; only one affected tooth per patient was included, and wisdom teeth were excluded. Teeth receiving initial conservative treatment were followed longitudinally in the registry until extraction, emigration, death, or the end of follow-up in 2023. Descriptive statistics, Kaplan–Meier survival analysis, and Cox regression models were applied to evaluate treatment patterns and factors associated with tooth survival.

**Results:**

In total, 14,881 teeth with ECR were identified (annual range: 1,132–2,071), of which 42.8% were extracted as the initial treatment and 57.2% received an initial conservative treatment (51.9% female; mean age 60.7 years, standard deviation [SD] 16.7; range 19–99), with or without surgery. The cumulative survival of conservatively treated teeth was 82.7% (standard error [SE] 0.4%) at 5 years, 74.9% (SE 0.5%) at 10 years, and 67.8% (SE 2.1%) at 15 years. In multivariate analyses, increasing age, fewer remaining teeth, molar involvement, and surgical treatment were associated with a higher risk of extraction, whereas root canal treatment performed within ±90 days of the initial treatment had no significant impact on tooth survival.

**Conclusion:**

In conclusion, this nationwide registry-based study provides population-level, long-term survival estimates for teeth affected by ECR and managed in routine dental practice. Although interpretation is limited by the lack of detailed clinical information inherent to registry data, the findings offer real-world evidence suggesting that teeth selected for conservative management demonstrate favourable long-term survival.

**Supplementary Information:**

The online version contains supplementary material available at https://doi.org/10.1186/s12903-026-09359-3.

## Background

External cervical resorption (ECR) has diverse clinical presentations and possible aetiologies, ultimately causing irreversible loss of hard tooth substance through clastic activity of the periodontal tissues [1]. They originate below the gingival attachment and progress in a coronal–apical direction, forming resorption channels and often stopping short of the vital pulp due to the pericanalar resorption-resistant sheet, with subsequent repair by bone-like tissue sometimes occurring [2]. ECR tends to be more aggressive in root-filled teeth, with less repair [3], and typically affects single teeth, although multiple teeth can be involved in some individuals [4–6]. It contrasts with internal resorptions, which arise within the pulp.

Several treatment options with varying degrees of complexity have been proposed. The therapy and its prognosis seem to depend on its severity [7]. Conservative therapy aims to halt lesion progression by mechanically removing the damaging tissue, with or without chemical assistance, and restoring the lost structure using various reparative materials to retain the tooth [1, 8]. Conservative therapy can be categorized based on the approach used to access the lesion: external repair (with or without flap surgery), internal repair (typically using the same access as for root canal treatment), or, if necessary, a combination of both [9]. Root canal treatments might be necessary to address coexisting inflammation or infection of the pulp space or to access the lesion. More complex approaches, such as surgical reimplantation or orthodontic extrusions, have also been reported [10, 11].

The evidence for the outcomes of these active conservative treatments is based on a few retrospective studies in highly specialized centres, reflecting the rarity and heterogeneity of the condition and the difficulty of conducting large clinical studies [5, 7, 12–16]. These studies typically include limited numbers of patients and may not be representative of outcomes achieved in routine dental practice, where probably most cases are managed and often report relatively short average follow-up periods (up to five years), leaving long-term outcomes at the group level insufficiently characterized [17]. Additionally, case reports illustrate potential treatment approaches but predominantly report favourable outcomes and therefore provide limited information on treatment effectiveness at the population level [8].

From a public health perspective, however, assessing disease burden, long-term tooth retention, and treatment patterns in real-world settings remains important, even for conditions of relatively low prevalence, not only to understand prognosis but also to place potential societal impact into context, including implications for resource use and clinical management outside highly specialized settings, where heroic treatment approaches—sometimes of limited relevance to routine clinical practice—are reported [18]. Large-scale longitudinal register data therefore provide a complementary approach by capturing care delivered in everyday practice and following affected teeth over long periods time, enabling population-based estimates of tooth survival that are not attainable from single-centre clinical cohorts.

Established in 2008, the Swedish National Dental Health Registry (SNDHR) provides access to large amounts of data that can be used to assess the effectiveness of these procedures in real-life settings, albeit with limited clinical detail available for each individual, as the registry does not include clinical records or radiographs (e.g., lesion extent) [19, 20]. The registry allows for the reporting of ECR by professionals as well as the treatments performed on those teeth associated with the diagnosis and tracks the teeth over time. Using such a registry might provide valuable insights into ECR that complement evidence derived from clinical studies.

Using data from the Swedish SNDHR, this study maps treatment modalities applied to teeth diagnosed with ECR in routine dental practice. The primary aim is to assess long-term tooth survival following treatments intended to preserve affected teeth at the population level, and the secondary aim is to explore associations between tooth survival and available variables within the constraints of registry data.

## Methods

The study was approved by the Swedish Ethical Review Authority (Dnr 2022–05579-01). The study was conducted in accordance with the Declaration of Helsinki (75th WMA General Assembly, Helsinki, October 2024). According to Swedish regulations, informed consent was not required for studies using anonymized registry data and was waived by the Swedish Ethical Review Authority. The guidelines for Strengthening the Reporting of Observational Studies in Epidemiology (STROBE) were followed when writing this report [21].

SNDHR was used to identify a nationwide cohort of all adult patients with registered tooth cavities due to external resorption from 2009 to 2018 and tracked treatments and outcomes longitudinally in the registry until 2023.

### Registry

The SNDHR is derived from data provided by the Swedish Social Insurance Agency (SSIA), which encompasses dental treatment records for all adults (>19 years) in Sweden, as all residents are insured under the SSIA. The register is a professionally maintained database with institutional oversight, including accreditation procedures, automated data validation, and post-payment audits. Reporting to the SSIA is restricted to licensed dental care practitioners, including general practitioners and specialists. As most practitioners are affiliated with the agency, nearly all interventions are systematically recorded. Notably, the rules governing benefits have evolved over time. All changes to the codes (conditions and treatment codes) are accessible at husk.tlv.se. One relevant change for this study was the increase in the age for free dental care for children and young adults, from 19 to 23 years old, in 2017. As a result, dental care records for patients aged 19–23 were no longer reported to the registry after 2017 and were therefore unavailable for this study. No individuals under 19 are represented in the database.

Each condition code is associated with only a limited set of approved treatment codes relevant to the specific condition. The target condition was code 4074: "tooth cavity due to external resorption”. Minor adjustments to the permitted treatments for code 4074 occurred during the recruitment period, but the treatment categories relevant to the present study remained unchanged; details are available in the national coding manual (husk.tlv.se). The treatment codes associated with code 4074 were tooth extraction codes, cavity filling codes, surgical codes (e.g., "other surgery or plastic surgery" and "periodontal surgical treatment for one to two teeth"), as well as supportive therapies (e.g., nitrous oxide sedation and healthcare contact to ensure proper coagulation). Codes could be combined (e.g., cavity filling codes with surgical codes) for the same caregiver on the same day on the same tooth position.

The registry also includes data on death and emigration dates as well as information on the number of remaining and intact teeth for each patient. Following prior validation of the registry, records reporting indicating either 32 intact teeth or zero remaining teeth were excluded, while the remaining data were retained as appropriate for analysis [22]. Missing data were limited to these exclusions and were not imputed.

### Recruiting in the database

The SNDHR was queried to identify all adult patients with a recorded diagnosis of condition code 4074 affecting any tooth between 1 January 2009 and 31 December 2018. In patients with more than one affected tooth, only the tooth with the earliest recorded diagnosis was included to ensure independence of observations and to avoid clustering at the patient level. Wisdom teeth were excluded. The treatment code recorded in conjunction with condition code 4074 was defined as the initial treatment. Teeth without a registered extraction or conservative treatment code associated with the initial diagnosis were excluded.

### Terminal events

Terminal events were defined as tooth extraction codes or extractions using a proxy code for extractions (e.g., pontic, implant, or removable prosthesis on the same tooth position). When terminal events coincided with the initial treatment, they were considered extraction as first treatments. Extraction codes were linked to the approved condition code recorded for that treatment episode.

### Observation period

Teeth were followed longitudinally from the date of initial treatment to a terminal event in the registry. If no such event occurred, the follow-up was censored at the patient's emigration, death, or on 31 December 2023. Initiated or completed root fillings (i.e., root canal treatments) within ±90 days of the initial treatment date were recorded. The presence of any additional treatments was registered.

### Variables and analysis

Descriptive statistics were employed to examine the frequencies of different therapies, including whether extraction was the first treatment or if other approaches aimed at preserving the tooth, referred to as conservative initial treatments, were used. Survival analyses were conducted to quantify the distribution of survival times for teeth treated with initial conservative therapies during the observation period.

In addition to overall tooth survival, the registry permitted the assessment of the following variables: sex; age; number of intact teeth; number of remaining teeth; tooth type of the resorbed tooth (defined as maxillary or mandibular incisors and canines, premolars, and molars); and type of initial conservative treatment (surgical or non-surgical based on the treatment codes).

Cox regression analyses (univariate and multivariate) were performed to assess the impact of these variables on tooth survival. All variables under study were included in the multivariate Cox regression model; none were excluded. All statistical analyses were performed using IBM SPSS Statistics for Windows, version 29.0.1.0 (IBM Corp., Armonk, NY, USA). A sample size calculation was not performed, as the expected number of cases was anticipated to be in the order of several thousand and there was no ethical reason to limit it.

## Results

### All teeth

After initial data cleaning, 14,881 individuals – 50.6% female and 49.4% male, with a mean age of 59.91 years (standard deviation [SD] 17.30; range 19–99) – were identified as having a registered tooth with a cavity due to external resorption. All tooth types were affected with the highest frequency in maxillary incisors and canines (*n* = 3,659, 24.6%) and the lowest in maxillary pre-molars (*n* = 1,257, 8.4%). The distribution between jaws was similar (50.4% maxillary). The highest frequency of included patients with ECR was recorded in 2009 (*n* = 2,071, 13.9%), with a steady decline each year, reaching the lowest in 2018 (*n* = 1,132, 7.6%).

### Tooth extraction as first treatment

A total of 6,366 teeth (42.8%) were extracted as a first treatment (49.1% female, 50.9% male; mean age 58.9 years, SD 18.4; range 19–98). The distribution of tooth types and the yearly prevalence of extractions closely mirrored the proportions observed for all teeth with the condition code 4074.

### Conservative initial treatments

After excluding seven patients with no conservative treatments registered, we identified 8,508 (57.2%) teeth with an initial conservative treatment. Of these, 51.9% were females (mean age: 60.7, SD 16.7, range 19–99). The distribution of tooth types and yearly prevalence of initial conservative treatments had similar proportions and trends observed for all teeth with the condition code 4074. Surgical procedures were performed on 779 (9.2%) teeth, while simpler cavity filling treatments (without surgery) were carried out on the remaining 7,729 (90.8%) teeth. Root canal treatments within ±90 days of the initial treatment date were performed in 1,366 (16.1%) teeth. Additional conservative treatments were reported in 3,493 (41.1%) teeth during the rest of the observation period. The mean observation time for teeth without terminal events was of 3,435 days (i.e., 9.4 years) (SD 1248 days, range 1–5,471).

Terminal events for teeth with initial conservative treatments were reported in 2,023 (23.8%) cases, with registered extractions accounting for 1,950 (96.4%) of these teeth. The associated registered conditions to extractions are shown in Table 1.

**Table 1:** Frequencies of the different diagnoses associated with known extractions of teeth that underwent initial conservative treatments

Grouped condition codes for teeth with initial conservative treatments, associated with extraction codes	Frequency (%)
Endodontic infections	648 (33.2%)
Periodontitis	496 (25.4%)
Root and/or crown fractures	403 (20.7%)
Resorption	209 (10.7%)
Caries	160 (8.2%)
Prosthetic failures	25 (1.3%)
Other	9 (0.5%)
Total	1950 (100%)

**Fig. 1:**
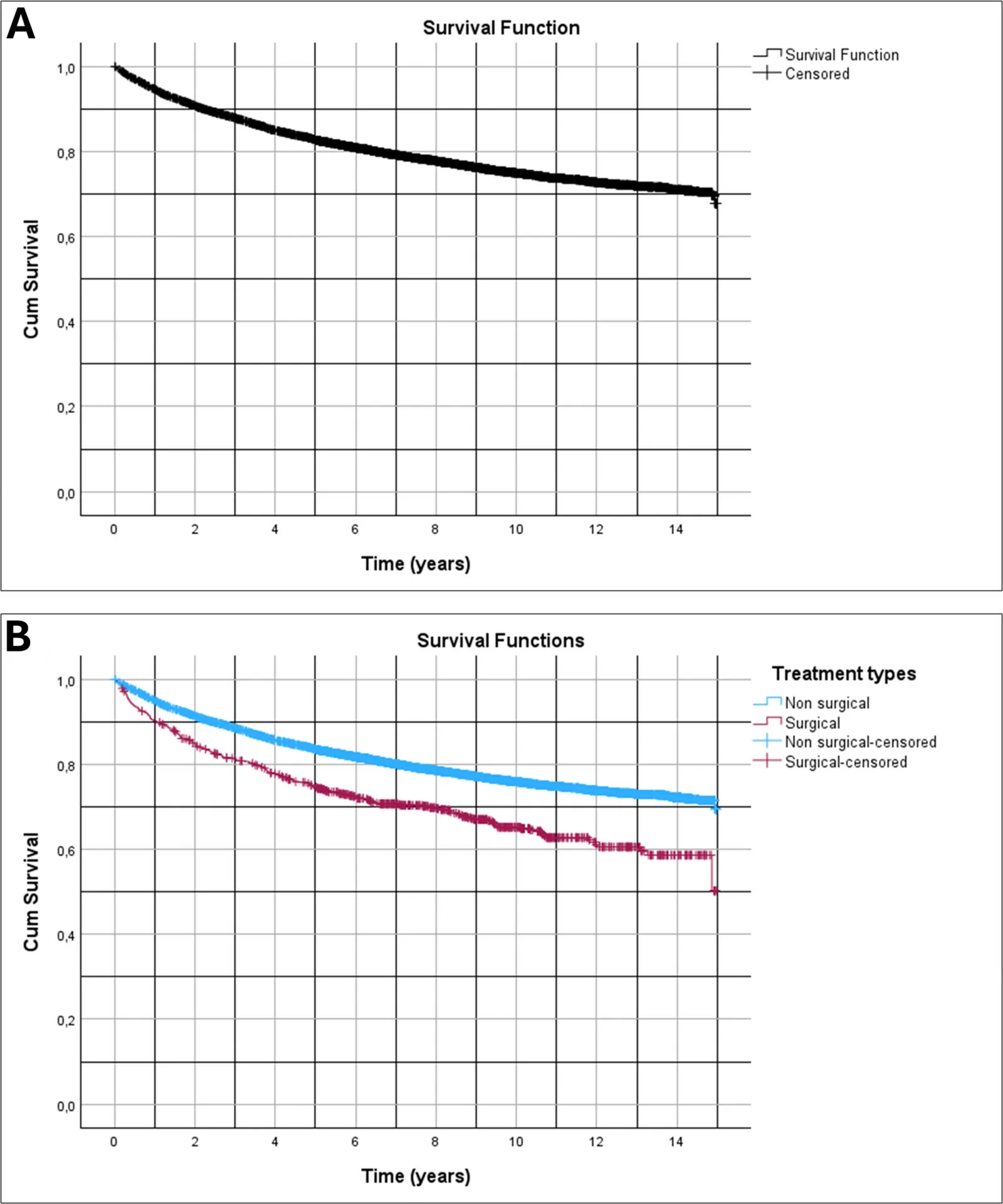
Kaplan-Meier survival curves: A = overall and B = treatment types

Kaplan-Meier plots are shown in Figure 1 for overall results and treatment choices. The survival table corresponding to the overall survival is shown in Table 2. The calculated 5-, 10-, and 15-year survivals were 82.7% (standard error [SE] 0.4%), 74.9% (0.5%), and 67.8% (SE 2.1%), respectively.

**Table 2:** Kaplan-Meier survival estimates and cumulative terminal events for teeth with initial conservative treatments

Year (end of period)	Cumulative survival	SE	Terminal events (cumulative)	Remaining teeth
1	94.6%	0.2%	462	7989
2	90.7%	0.3%	782	7583
3	87.9%	0.4%	1022	7235
4	85.0%	0.4%	1259	6876
5	82.7%	0.4%	1439	6565
6	80.8%	0.4%	1580	5784
7	79.2%	0.5%	1692	5052
8	77.8%	0.5%	1778	4340
9	76.2%	0.5%	1857	3653
10	74.9%	0.5%	1915	3047
11	73.7%	0.5%	1959	2434
12	72.8%	0.6%	1988	1848
13	71.9%	0.6%	2007	1218
14	71.0%	0.6%	2017	644
15	67.8%	2.1%	2023	0

*Abbreviations*: *SE* Standard error

Cox-regression analyses are shown in Table 3 (univariate, together with baseline characteristics and distribution of terminal events) and Table 4 (multivariate analyses). Root filling performed within 90 days before or after the initial treatment had no impact on the outcome (*p* = 0. 236). In the univariate analysis of all analysed variables, only sex showed no association with the outcome. In the multivariate analysis, the effect of the number of intact teeth was no longer significant as well. The factors that remained statistically significant and associated with a higher hazard for a terminal event were increasing age, fewer remaining teeth, molars, and surgical treatments (Figure 1, Table 4); however, the associations should be interpreted cautiously because important clinical variables, such as lesion extent, anatomical complexity, and restorability, were unavailable.

**Table 3:** Baseline characteristics of teeth diagnosed with ECR, distribution of terminal events and univariate regression analyses

Variables	Total (n [%] or mean ± SD)	Terminal Events (n), %	Hazard ratio	95% confidence interval	*P* value
Sex					
Male (0)	4096 (48.1%)	961 (23.5%)			
Female	4412 (51.9%)	1062 (24.1%)	1.031	0.944–1.125	0.499
Age	n=8508 60.7 ± 16.7	2023 (23.8%)	1.018	1.016–1.021	<0.001
Number of remaining teeth (range 1–32)	n=8246 25.6 ± 5.0	1972 (23.9%)	0.959	0.952–0.967	<0.001
Number of intact teeth (range 0 −31)	n=8246 11.4 ± 8.4	1972 (23.9%)	0.973	0.968–0.979	<0.001
Tooth type					<0.001
maxillary incisors and canines (0)	1909 (22.4%)	337 (17.7%)			
maxillary pre-molars	812 (9.5%)	159 (19.6%)	1.124	0.931–1.357	0.226
maxillary molars	1400 (16.5%)	409 (29.2%)	1.858	1.609–2.147	<0.001
mandibular incisors and canines	1324 (15.6%)	320 (24.2%)	1.553	1.333–1.810	<0.001
mandibular pre-molars	1294 (15.2%)	300 (23.2%)	1.462	1.252–1.709	<0.001
mandibular molars	1769 (20,8%)	498 (28.2%)	1.735	1.511–1.993	<0.001
Type of treatment					
Non-surgical (0)	7729 (90.8%)	1765 (22.8%)			
Surgical	779 (9.1%)	258 (33.1%)	1.607	1.410–1.831	<0.001

*Abbreviations*: *SD* Standard deviation

**Table 4:** Multivariate Cox-regression model for tooth survival following initial conservative treatment of ECR

Variables	Hazard ratio	95% confidence intervals	*P* value.
Sex (Male = reference)	1.036	0.947–1.312	0.441
Age	1.027	1.010–1.044	0.002
Number of remaining teeth	0.971	0.961–0.981	<0.001
Number of intact teeth	0.997	0.989–1.005	0.793
Tooth type			
maxillary incisors and canines (0)			
maxillary pre-molars	1.133	0.936–1.372	0.201
maxillary molars	1.915	1.652–2.219	<0.001
mandibular incisors and canines	1.175	1.002–1.379	0.048
mandibular pre-molars	1.220	1.040–1.431	0.015
mandibular molars	1.744	1.514–2.008	<0.001
Type of treatment (Non-surgical = reference)	1.470	1.286–1.681	<0.001

## Discussion

Rather than treating with extractions, the teeth with external resorption were treated conservatively in the majority of cases (57.2%). The survival rates for these conservative treatments were considerably high over prolonged periods. The majority of registered treatments were non-surgical with simple fillings.

## Methods

The rationale for inclusion and exclusion was straightforward. Wisdom teeth were excluded due to the common occurrence of resorption in impacted or semi-erupted teeth, which poses a different clinical scenario. Although exclusion of second molars was considered (i.e., resorptions may be caused by erupting wisdom teeth and could be classified as external surface resorption), they were ultimately included as the lesions are frequently located cervically. A sensitive analysis did not show any change in the overall survival curve when second molars were removed. Only the first affected tooth per patient was included to avoid clustering and maintain independence of observations. Consequently, outcomes from additional affected teeth in patients with multiple lesions were not captured, which may have influenced the reported treatment frequencies and survival estimates to an unknown extent.

The recruitment period served two purposes: to generate a robust dataset and to ensure a minimum observation period of five years for all potential surviving teeth. Tooth extraction, the chosen outcome, is patient-centred and easily tracked through the registry which does not allow assessment of other biological success measures.

As with all register-based research, the validity of findings depends on the accuracy and consistency of recorded diagnostic codes rather than on direct clinical verification, which warrants careful consideration when interpreting results derived from routinely collected data. In the absence of condition-specific validation of registry codes for ECR, a degree of diagnostic uncertainty is inherent to register-based research. However, the SNDHR has demonstrated high reliability in economic reporting, with prior evaluations indicating 93.7% correct reimbursement claims and small degree of error in data registration [23]. Although these audits were not specific to the clinical variables used in the present study, there is substantial overlap in coding practices, supporting general confidence in data quality. The very small number of registered cases—in relation to ~16 millions of yearly SSIA submissions—suggests that the use of the ECR code is highly selective, making miscoding of more common conditions, such as caries, unlikely. This selective use of an uncommon, non-default diagnostic code could indicate a high degree of diagnostic specificity, as its application is likely confined to clinicians familiar with both the condition and the whole coding system. Moreover, the SSIA resorption code describes defects amenable to conservative restorative management, which closely reflects the typical clinical presentation of ECR and further supports specificity by naturally excluding other major types of tooth resorption. The only potential non-ECR overlap would be external surface resorption of second molars, which has been considered. Under-ascertainment, whereby ECR may be diagnosed or recorded under alternative codes, is therefore more likely than over-ascertainment and would be expected to primarily reduce the number of identified cases rather than systematically bias estimates of tooth survival. This selectivity should be considered when interpreting the findings, as it likely reflects variation in diagnostic and reporting awareness among clinicians, potentially introducing operator and observer bias.

Further support for the interpretability of registry-derived outcomes comes from previous studies based on the same national dental registry, which examined the survival of root-filled teeth. These registry-based investigations have reported survival estimates that correspond well with results from smaller retrospective clinical studies conducted in selected Swedish populations, in which detailed clinical records were reviewed over comparable timeframes. Importantly, although formal validation of diagnostic and treatment codes was not performed in these registry-based survival studies, the concordance of outcomes across registry-derived and clinically validated datasets suggests that survival estimates obtained from this registry are broadly comparable to those derived from detailed clinical data [20, 24, 25].

Register studies generate robust sample sizes and are particularly useful for investigating conditions with lower frequencies. In this context, professionally maintained health registries enable longitudinal follow-up of patient-centred outcomes within the general population in real-world clinical settings.

Taken together, these considerations indicate that while diagnostic misclassification cannot be entirely excluded in a register-based design, the resulting uncertainty is, in our interpretation, unlikely to have introduced relevant systematic bias in estimates of tooth survival; however, findings should be interpreted in the context of selective case ascertainment inherent to registry data.

Registries have additional inherent constraints. As this was a registry-based rather than a clinical study, data collection was not controlled by the researchers, and analyses were necessarily restricted to information available within the registry. The SNDHR contains coded data on diagnoses and overall treatments only and does not include clinical records, radiographs, or procedural documentation; consequently, key confounders and clinically important variables—such as lesion severity classification, tooth restorability, procedural details, operator identity, assessment of biological success—were not recorded and could not be included in the analyses. Consequently, unmeasured factors may have introduced residual confounding, and findings related to the secondary aim should be considered exploratory, rather than evidence of treatment superiority or inferiority [26]. These constraints limit the clinical inferences that can be drawn from the study; however, the registry provides important population-level information on treatment patterns and long-term outcomes in routine dental practice that cannot be obtained from clinical cohorts. Future studies may benefit from linkage with other Swedish national registers to incorporate additional socioeconomic and health-related variables that were unavailable in the present dataset.

## Results

The study could not capture monitoring approaches, as the registry does not contain specific monitoring codes and clinical records were unavailable; when lesions are asymptomatic and clinically undetectable monitoring is considered effective [12]. Cases associated only with supportive treatment codes were excluded because no definitive treatment strategy could be determined. Furthermore, variation in diagnosis, reporting, and registration practices may result in under-ascertainment of ECR cases. Therefore, the study cannot provide accurate annual incidence rates; however, based on the number of registered cases over the study period, a minimum annual incidence can be estimated at >15 patients per 100,000 adults per year, using the Swedish adult population of approximately 9.5 million as the denominator. Although incidence and prevalence are not directly comparable measures, the prevalence of ECR in patients has been estimated to range between 0.6% and 8% [27–30]. However, these studies, based on CBCT for clinical indications—often endodontic—are subject to sampling bias and tend to overestimate prevalence, whereas a population-based Danish study of 17-year-olds reported ECR in ~0.2% [31].

The results indicate an encouraging survival rate for teeth registered with external resorptions. However, the robustness of the data from the end of the period is somewhat compromised by a relatively smaller remaining population. At first glance, the tooth survival rate in the present study appears significantly better than in the largest study to date [12], which included over 300 patients at baseline, and reported <30% survival at 10 years. Their results are most probably biased by substantial loss to follow-up—75% by 10 years—and the inadequate use of simple proportions that ignored censored data, likely underestimating survival [32]. If similar methods were applied here, the survival curve would show a comparable decline toward the end of the observation period, driven by a decreasing number of individuals remaining under observation as loss to follow-up increases (Supplement 1).

In contrast, based on Mavridou et al.’s data [12], the extraction rate in the baseline cohort was 17.7% (60 of 339 teeth), which is comparable to the 22.9% extraction rate observed among teeth with initial conservative treatments in the present study, or 23.8% when all terminal events were considered.. The slightly higher proportion observed here is likely attributable to the substantially longer mean observation period. Across all known cohort studies, 135 of 773 teeth at baseline (17.5%) were extracted over 1–12 years [5, 7, 12–16]. Although, these comparisons do not account for patient loss or varying follow-up times, they are broadly consistent with the present findings when interpreted in the context of their shorter observation period. Kaplan–Meier curves reported by Mavridou et al. (Supplement) align closer with our findings, and a recent study has similarly reported censored survival curves with up to 5 years of follow-up, showing similar results [16]. Building on earlier descriptive reports, the present study applies Kaplan–Meier survival analysis to demonstrate that outcomes are consistent with previously published findings within the shorter follow-up periods available in the literature, where loss to follow-up is less pronounced. The study further extends these observations by providing survival estimates with formal uncertainty measures over longer time frames of up to 15 years, thereby offering the most comprehensive longitudinal assessment of tooth survival in ECR reported to date. This information is highly relevant not only for clinicians making treatment decisions, but also for other stakeholders concerned with population-level oral health outcomes.

In this study, 42.8% of teeth were extracted as initial treatment, higher than in previous reports [12, 15]. This pattern may reflect a clinically sound and pragmatic case-selection strategy in routine practice, whereby less complex lesions are preferentially treated, while very extensive lesions with a known poor prognosis are less often pursued, partly explaining the favourable outcomes observed [16]. Extractions following conservative treatment had multiple possible indications, yet only 10.7% were attributed to resorptions. As dentists can report only one diagnosis, determining whether tooth loss was a competing risk or related to the lesion is challenging. In this cohort, endodontic infections, periodontitis, fractures, and other diagnoses were commonly recorded in association with extraction (Table 1). All extractions were considered terminal events.

Interpretation of the observed associations should be cautious, as the registry as expected lacked many variables, and unregistered confounders—such as lesion severity, anatomical complexity, and restorability—may influence results. Certain patient-related factors were associated with overall tooth survival, but treatment comparisons and differences between tooth types are biased by selection and reflect differing clinical scenarios rather than true superiority. The associations observed for age and number of remaining teeth are not unexpected and likely reflect broader oral health status and the cumulative burden of conditions leading to tooth loss over time, rather than factors specific to ECR. Given the limited clinical information available in the registry, further interpretation would be largely speculative, and these findings should therefore be considered exploratory. Most treatments were non-surgical, likely performed on earlier-stage lesions, which may explain the favourable outcomes. Accordingly, the observed association between surgical treatment and earlier tooth loss should not be interpreted as evidence that surgical treatment is less effective than non-surgical treatment. These findings contrast with Irinakis et al. [14], who found more favourable outcomes for teeth with root canal treatments.

## Conclusions

A substantial proportion of teeth diagnosed with ECR were extracted as an initial treatment approach in routine dental practice. At the population level, the results indicate that teeth selected for preservation through surgical or restorative (non-surgical) procedures show favourable long-term survival (up to 15 years). Factors such as older age, reduced dentition, molar involvement, and surgical treatment were associated with earlier tooth loss; however, these associations may be influenced by unmeasured clinical variables, such as lesion extent, and should therefore be interpreted cautiously and further explored in future clinical studies with more detailed patient- and lesion-level data. As a registry-based study, the findings provide a broad real-world overview of treatment patterns, disease burden as reflected by actually treated cases, and long-term outcomes in routine dental practice, but should be interpreted in light of the inherent limitations of routinely collected data and the lack of detailed clinical information.

## Supplementary Information

Supplementary Material 1. Supplement 1. Series 1 illustrates the survival curve calculated from the proportion of teeth known to remain *in situ* over time relative to the total number of teeth with known outcomes, using the data shown in Table 2. It is the same method applied by Mavridou et al. [12]. Series 2 reflects the best possible scenario, calculated taking into consideration the known extractions at each year-end relative to the total population of 8,508 teeth, rather than only those with known outcomes. This curve assumes no additional tooth extractions in patients lost to follow-up, which is unrealistic. Series 3 is a reconstructed Kaplan-Meier curve with values calculated only at the end of each year. By the 14^th^ year, the Kaplan-Meier curve shows a +46.8% absolute difference compared to Serie 1 and a −5.3% absolute difference compared to Series 2. In this material, the curves begin to diverge between years 5 and 6 as follow-up drops exceed 10%, with further divergence as losses increase.

## Data Availability

The data that support the findings of this study are available from the Swedish National Dental Health Registry, maintained by the Swedish Social Insurance Agency (SSIA), but restrictions apply to the availability of these data, which were used under licence for the current study and are therefore not publicly available. Data are however available to researchers who fulfil the criteria for access to confidential data upon reasonable application to the SSIA and with permission from the relevant ethical review authority.

## Abbreviations

CBCT: Cone-Beam Computed Tomography
ECR: External Cervical Resorptions
SD: Standard Deviation
SE: Standard Error
SNDHR: Swedish National Dental Health Registry
SSIA: Swedish Social Insurance Agency
