# Supplementary material for: External cervical resorption treatments in routine dental practice: a nationwide registry-based cohort study with long-term follow-up
Source: BMC Oral Health. 2026 Jul 23;26:1744. doi: 10.1186/s12903-026-09359-3 (PMC13576311; doi:10.1186/s12903-026-09359-3)
Supplement: Supplementary file 1 [file 12903_2026_9359_MOESM1_ESM.docx]

## Supplement

Cumulative survival

| Time (year) |  |  |  |
| --- | --- | --- | --- |
| 1 | 0.945332 | 0.945698 | 0.946 |
| 2 | 0.906515 | 0.908087 | 0.907 |
| 3 | 0.876226 | 0.879878 | 0.879 |
| 4 | 0.845237 | 0.852022 | 0.85 |
| 5 | 0.820215 | 0.830865 | 0.827 |
| 6 | 0.785443 | 0.814292 | 0.808 |
| 7 | 0.74911 | 0.801128 | 0.792 |
| 8 | 0.709382 | 0.79102 | 0.778 |
| 9 | 0.662976 | 0.781735 | 0.762 |
| 10 | 0.614067 | 0.774918 | 0.749 |
| 11 | 0.554063 | 0.769746 | 0.737 |
| 12 | 0.481752 | 0.766338 | 0.728 |
| 13 | 0.377674 | 0.764104 | 0.719 |
| 14 | 0.242014 | 0.762929 | 0.71 |
| 15 | 0 | 0.762224 | 0.678 |

Supplement 1. Series 1 illustrates the survival curve calculated from the proportion of teeth known to remain *in situ* over time relative to the total number of teeth with known outcomes (extracted or surviving), using the data shown in Table 2. It is the same method applied by Mavridou et al. [12]. Series 2 reflects the best possible scenario, calculated taking into consideration the known extractions at each year-end relative to the total population of 8,508 teeth, rather than only those with known outcomes. This curve assumes no additional tooth extractions in patients lost to follow-up (shorter observations), which is unrealistic. Series 3 is a reconstructed Kaplan-Meier curve with values calculated only at the end of each year. By the 14^th^ year, the Kaplan-Meier curve shows a +46.8% absolute difference compared to Serie 1 and a −5.3% absolute difference compared to Series 2. In this material, the curves begin to diverge between years 5 and 6 as follow-up drops exceed 10%, with further divergence as losses increase.
